# SIRT1 attenuates renal fibrosis by repressing HIF-2α

**DOI:** 10.1038/s41420-021-00443-x

**Published:** 2021-03-23

**Authors:** Peipei Li, Yue Liu, Xiaogang Qin, Kairen Chen, Ruiting Wang, Li Yuan, Xiaolan Chen, Chuanming Hao, Xinzhong Huang

**Affiliations:** 1 Department of Nephrology, Affiliated Hospital of Nantong University, 20 Xisi Road, 226001 Nantong, Jiangsu China; 2 Department of Nephrology, Traditional Chinese Medicine Hospital of Tongzhou District, Nantong, 8 Jianshe Road, 226300 Nantong, Jiangsu China; 3 Division of Nephrology, Huashan Hospital, and Nephrology Research Institute, Fudan University, 12 Urumqi Middle Road, Shanghai, China

**Keywords:** Chronic kidney disease, Kidney

## Abstract

Sirtuin 1 (SIRT1) is a nicotinamide adenine dinucleotide (NAD^+^)-dependent deacetylase belonging to class III histone deacetylases. Previous studies have shown that SIRT1 is involved in kidney physiology regulation and protects the kidney from various pathological factors. However, the underlying mechanisms behind its function have yet to be fully elucidated. In our study, we found that ablation of *Sirt1* in renal interstitial cells resulted in more severe renal damage and fibrosis in unilateral ureteral obstruction (UUO) model mice. We also observed that hypoxia-inducible factor (HIF)-2α expression was increased in *Sirt1* conditional knockout mice, suggesting that HIF-2α might be a substrate of SIRT1, mediating its renoprotective roles. Therefore, we bred *Hif2a* deficient mice and subjected them to renal trauma through UUO surgery, ultimately finding that *Hif2a* ablation attenuated renal fibrogenesis induced by UUO injury. Moreover, in cultured NRK-49F cells, activation of SIRT1 decreased HIF-2α and fibrotic gene expressions, and inhibition of SIRT1 stimulated HIF-2α and fibrotic gene expressions. Co-immunoprecipitation analysis revealed that SIRT1 directly interacted with and deacetylated HIF-2α. Together, our data indicate that SIRT1 plays a protective role in renal damage and fibrosis, which is likely due to inhibition of HIF-2α.

## Introduction

Chronic kidney disease (CKD) is a general term that encompasses multifarious disease pathways leading to irreversible changes in kidney function and structure over the course of months or years^1^. CKD is a serious public health problem characterized by poor health outcomes and extraordinarily high healthcare costs^2,3^. Renal fibrosis is a chronic and progressive process that alters kidney function and structure, serving as the shared final pathway of nearly all CKD conditions, irrespective of cause^4^. However, effective treatments to delay renal fibrosis progression and prevent or ameliorate CKD-related complications are quite limited. As such, there is an urgent need for more research about the molecular mechanisms underlying the progression of renal fibrosis.

Sirtuin 1 (SIRT1) is a nicotinamide adenine dinucleotide (NAD^+^)-a dependent enzyme with numerous pleiotropic effects, including metabolic, oxidative, and hypoxic stress resistance; DNA repair, and longevity^5^. Previous studies have shown that SIRT1 is expressed in the kidney and plays beneficial roles in renal physiology and pathology^6–9^. SIRT1 has been shown to have antifibrotic functions in several animal models. For example, He et al. found that SIRT1 prevents kidney tissue from engaging in fibrogenesis by regulating oxidative stress-induced COX2 expression in UUO model mice^6^. Furthermore, our previous results showed that, by inhibiting the transcriptional activity of Smad3, SIRT1 plays a protective role against renal fibrosis in a CKD rodent model^10^.

In the kidney, renal interstitial cells are responsible for constructing the extracellular matrix (ECM)^11^. Recently, a growing amount of evidence suggests that renal interstitial cells play a vital role in the development of renal fibrosis^12^. Normally, the synthesis and degradation of ECM are in dynamic equilibrium. However, under pathological conditions, fibrotic factors activate renal interstitial cells, leading to the accumulation of excessive ECM accompanied by morphological changes, thus causing renal fibrosis^12^. To investigate this, we bred SIRT1 conditional knockout mice and examined whether SIRT1 deficiency in renal interstitial cells affects renal fibrosis.

Multiple studies have emphasized that renal tissue hypoxia is closely connected to the progression of CKD, considering it to be a probable initiating event and final common pathway in the process^13–15^. It is well-known that hypoxia-inducible factor (HIF) is a major transcription factor that adapts hypoxia responses in numerous pathophysiological processes of kidney diseases^16–18^. In the kidney, HIF-1α and HIF-2α are the two main isoforms of HIF, with different expression sites. HIF-1α is mainly expressed in tubular epithelial cells, whereas HIF-2α expression is induced by hypoxia in peritubular endothelial cells and interstitial cells^19^.

In this study, we found that SIRT1 plays a protective role against renal damage and fibrosis. Deletion of *Sirt1* in renal interstitial cells resulted in more severe renal fibrosis in UUO model mice. Meanwhile, HIF-2α expression was induced after UUO, especially in *Sirt1* deficient mice. Specific ablation of *Hif2a* in renal interstitial cells attenuates renal fibrosis induced by UUO surgery. As such, we conclude that the deletion of *Sirt1* aggravates renal damage and fibrosis, which may depend upon activation of HIF-2α.

## Results

### SIRT1 expression is increased in kidneys from UUO model mice

**Fig. 1:**
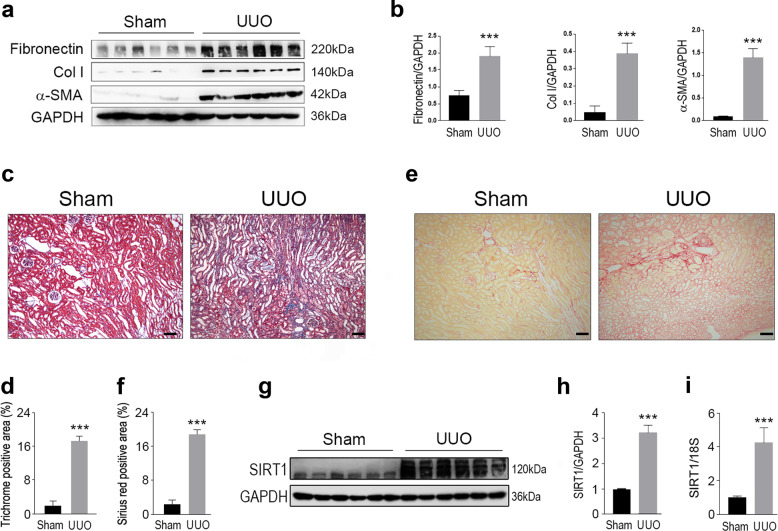
SIRT1 is upregulated in the kidney from UUO modeled mice. Male wild-type C57BL/6 mice, at 8–10 weeks of age, were subjected to UUO operation. Mice were euthanized 10 days after sham or UUO surgery. **a** Western blot analysis showed that fibronectin, collagen I (Col I), and a-SMA in the kidney were increased upon UUO surgery. GAPDH was used as a loading control. **b** Quantification of western blot data as shown in **a**. **c** Masson-trichrome staining. Scale bar = 50 μm. **d** Quantitative analysis for **c**. **e** Sirius red staining. Scale bar = 50 μm. **f** Quantitative analysis for **e**. **g** SIRT1 in the kidney was enhanced in UUO-operated mice. Protein levels were analyzed by western blot analysis and GAPDH has used a loading control. **h** Quantification of western blot data as shown in **e**. **i** SIRT1 mRNA levels were increased in the kidney from UUO mice. *n* = 6. Data are means ± SEM. ***P < 0.001.

To investigate the potential roles of SIRT1 in kidney lesions, we bred UUO model mice, which are known to be suitable subjects in which to induce renal fibrosis. Masson’s trichrome and Sirius Red staining showed severe renal fibrosis in UUO mice (Fig. 1c-f), and as expected, the protein levels of fibronectin, collagen I (Col I), and α-SMA in the kidneys were markedly increased in UUO mice compared to those of normal mice (Fig. 1a, b). We also analyzed SIRT1 expression and found that the protein levels of SIRT1 in the kidneys were enhanced by UUO (Fig. 1g, h). In accordance with the changes in SIRT1 protein levels, mRNA levels were also increased in the kidneys of UUO mice (Fig. 1i). These data suggest that SIRT1 may be involved in the progression of renal fibrosis.

### *Sirt1* deficiency in interstitial cells aggravates renal damage in UUO mice

**Fig. 2:**
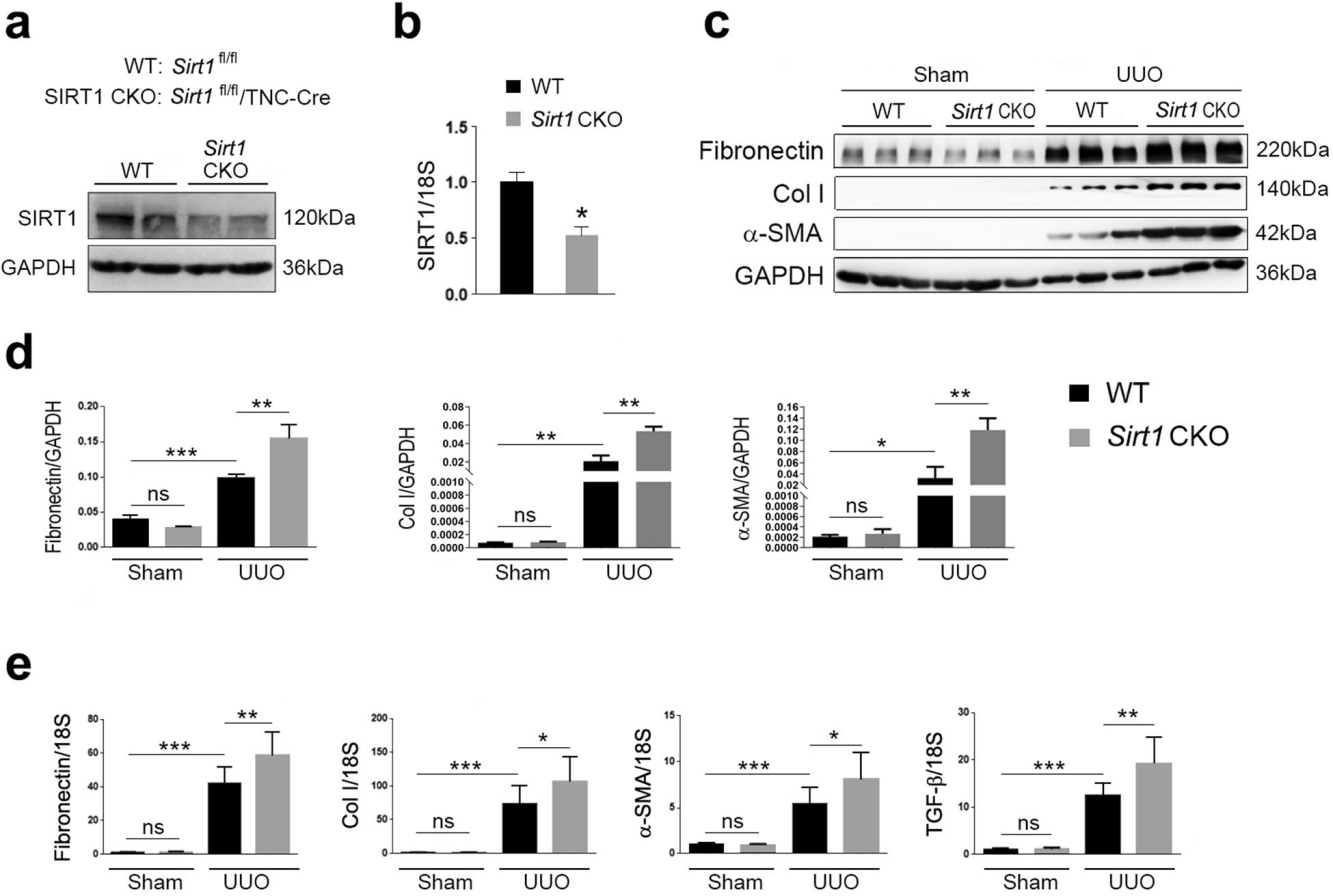
SIRT1 deficiency aggravates UUO injury in the kidney. **a** SIRT1 was decreased in the kidney in kidney conditional knockout mice (*Sirt1* CKO). Protein levels were analyzed by western blot analysis and GAPDH was used as a loading control. **b** SIRT1 mRNA levels in the kidney were also reduced in *Sirt1* CKO mice. **c** SIRT1 deficiency further exacerbates kidney fibrosis induced by UUO surgery. Protein levels were analyzed by western blot analysis and GAPDH was used as a loading control. **d** Quantification of western blot data as shown in **c**. **e** *Sirt1* deficiency in the kidney stimulates kidney fibrosis-related gene expressions in UUO mice. Gene expression was analyzed by qRT-PCR and 18S has used a house-keeping gene. *n* = 6. Data are means ± SEM. *P < 0.05, **P < 0.01, and ***P < 0.001. ns means no significance.

**Fig. 3:**
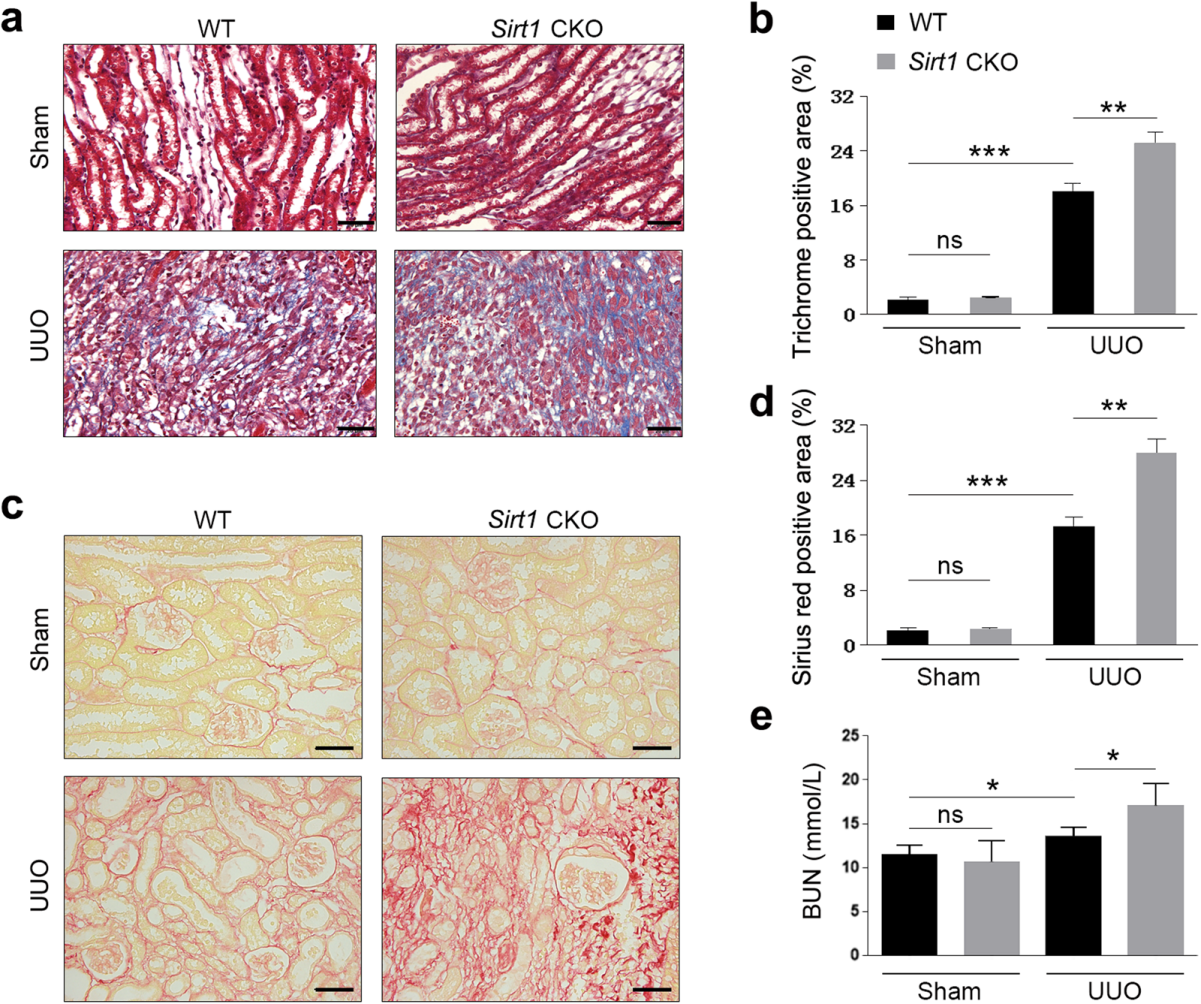
Renal interstitial cell-specific ablation of *Sirt1* enhances fibrogenesis during UUO injury. Male WT (*Sirt1*^flox/flox^) and *Sirt1* CKO (*Sirt1*^flox/flox/TNCCreER^), at 8–10 weeks of age, were subjected to UUO operation. Mice were sacrificed on the 10th day after sham or UUO surgery. **a** Paraffin section from kidneys of sham or UUO surgery mice was analyzed by Masson-trichrome staining. Scale bar = 20 μm. **b** Quantitative analysis of Masson-trichrome staining. **c** Sirius red staining. Scale bar = 20 μm. **d** Quantitative analysis of Sirius red staining. **e** BUN levels. *n* = 6. Data are means ± SEM; ns: no significance. *P > 0.05, **P < 0.01, ***P < 0.001.

To examine the potential roles of SIRT1 in renal fibrosis, we bred SIRT1 CKO mice by crossing floxed *Sirt1* mice with TNCCreER mice. This has been shown to induce DNA recombination; specifically, in the renal medullary interstitial cells of the kidney^20^. Therefore, the *Sirt1* CKO mice bred in this study had SIRT1 deficient renal interstitial cells. Littermates not carrying the Cre transgene served as control group animals (*Sirt1*^fl/fl^; wild-type [WT]). Protein levels of SIRT1, as well as mRNA levels, were dramatically reduced in the kidneys of *Sirt1* CKO mice (Fig. 2a, b). Once the above was ascertained, we performed UUO surgery in *Sirt1* CKO and WT mice. As we observed above, expression of renal fibrosis markers, including fibronectin, Col I, and α-SMA, was vastly increased in all kidneys after UUO surgery (Fig. 2c, d). Interestingly, *Sirt1* CKO mice experienced greater increases than their counterparts (Fig. 2c, d). We also measured the expression of these fibrosis marker genes at the mRNA level, and similar changes to their protein levels were observed (Fig. 2e). The expression of TGF-β was induced by UUO, which was further increased in *Sirt1* CKO mice (Fig. 2e). Renal damage induced by UUO was further aggravated by *Sirt1* deficiency, as evidenced by histological analyses (Fig. 3a–d). Blood urea nitrogen (BUN) levels were raised in UUO mice and were further increased by *Sirt1* deficiency (Fig. 3e). These data clearly show that *Sirt1* deficiency in interstitial cells further worsens renal fibrosis induced by UUO surgery, suggesting that SIRT1 may play a renoprotective role against renal fibrosis.

### *Sirt1* deficiency stimulates HIF-2α expression in the kidney

**Fig. 4:**
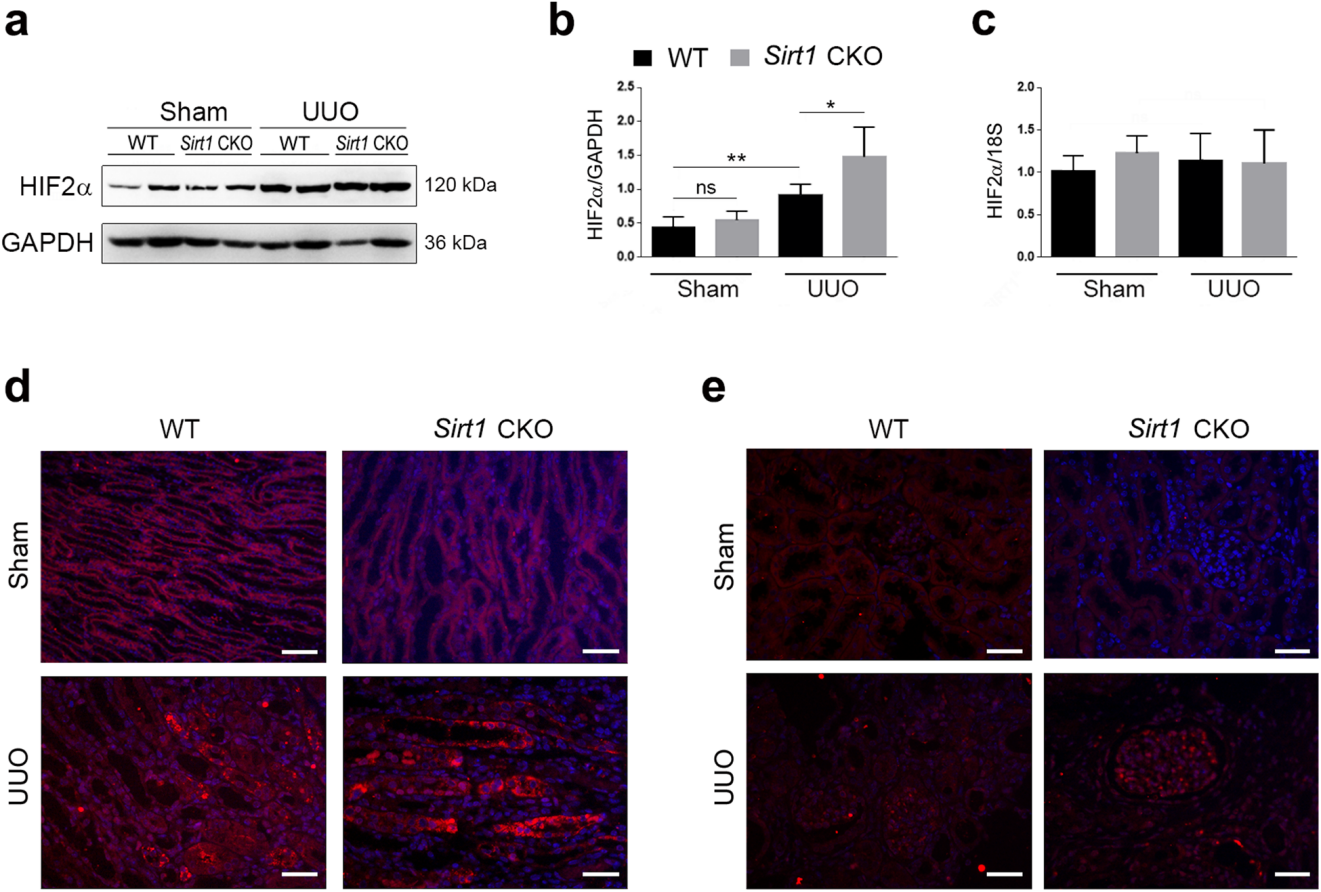
HIF-2α in the kidney is stimulated by UUO injury. **a** UUO injury increases HIF-2α protein levels in kidneys of WT and *Sirt1* CKO mice. Protein levels were analyzed by western blot analysis and GAPDH was used as a loading control. **b** Quantitative analysis of western blot data as shown in **a**. **c** HIF-2α mRNA levels were not altered by UUO surgery. Gene expression was analyzed by qRT-PCR and 18S was used as a house-keeping gene. **d**–**e** HIF-2α in the renal tubule (**d**) and glomerulus (**e**) were analyzed by immunofluorescence. Scale bar = 20 μm. *n* = 6. Data are means ± SEM. ns: no significance. *P > 0.05, **P < 0.01.

HIF-2α has been shown to participate in renal physiology and pathology. Therefore, in the following experiments, we focused our interest on the SIRT1/HIF-2α axis of renal fibrosis. To this end, we analyzed whether HIF-2α expression is altered by SIRT1 in sham and UUO mice. The results showed that HIF-2α protein expression was increased in the kidneys of all mice subjected to UUO surgery, with a greater increase in *Sirt1* CKO mice (Fig. 4a, b). However, the mRNA levels of HIF-2α were not affected by UUO surgery or *Sirt1* deficiency in either group (Fig. 4c). Immunostaining showed HIF-2α expression in the renal tubule and glomerulus was induced by UUO, especially in *Sirt1* CKO mice (Fig. 4d, e). Therefore, we predicted that HIF-2α may be an important factor in mediating *Sirt1* deficiency-induced renal damage and fibrosis.

### *Hif2a* deficiency in renal interstitial cells attenuates kidney damage in UUO mice

**Fig. 5:**
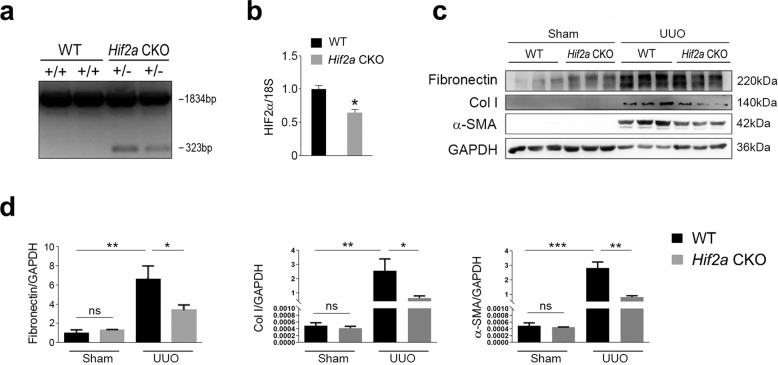
*Hif2a* deficiency in renal interstitial cells attenuates fibrogenesis induced by UUO injury. **a** Genotyping for *Hif2a* CKO mice. The length of PCR products for the wild-type *Hif2a* allele is 1834 bp, and the length for the knockout *Hif2a* allele is 323 bp. **b** HIF-2α expression was reduced in kidneys from *Hif2a* CKO mice. Gene expression was analyzed by qRT-PCR and 18S has used a house-keeping gene. **c** Renal fibrosis was attenuated by *Hif2a* deficiency. Protein levels were analyzed by western blot and GAPDH was used as a loading control. **d** Quantitative analysis of western blot data as shown in **c**. *n* = 6. Data are means ± SEM. ns: no significance. *P > 0.05, **P < 0.01, ***P < 0.001.

**Fig. 6:**
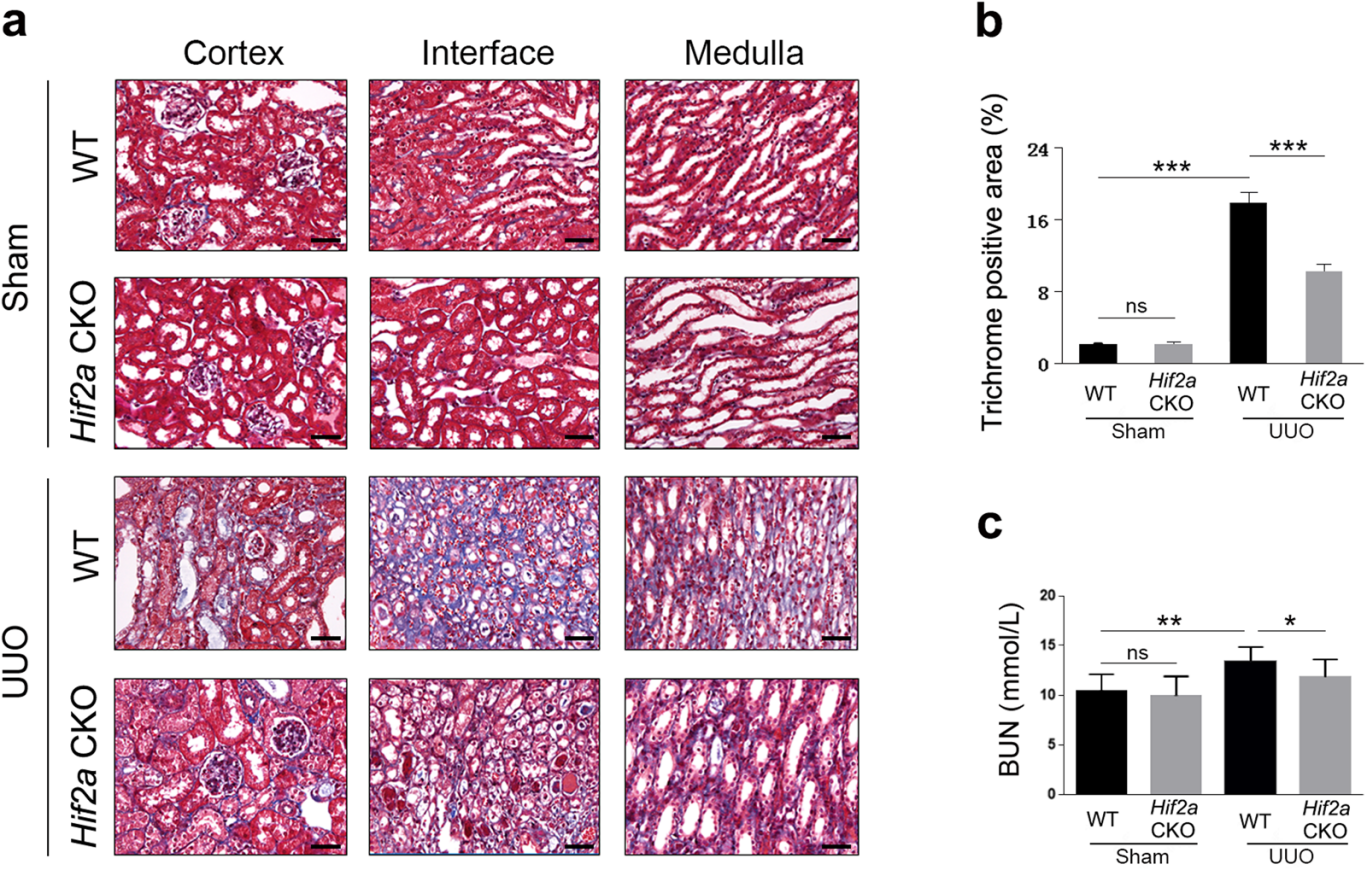
*Hif2a* deficiency ameliorates renal fibrosis induced by UUO surgery. **a** Masson-trichrome staining showing fibrosis was reduced. Scale bar = 20 μm. **b** Quantitative analysis of the data shown in **a**. **c** BUN levels was decreased in *Hif2a* CKO mice. *n* = 6. Data are means ± SEM. ns means no significance. *P > 0.05, **P < 0.05, ***P < 0.001.

To examine the potential role of HIF-2α in renal fibrosis, we generated *Hif2a* conditional heterozygous knockout mice (*Hif2a* CKO) with *Hif2a* deleted in their interstitial cells (confirmed by genotyping and qRT-PCR data) (Fig. 5a, b). These *Hif2a* CKO mice, together with WT mice, were then subjected to UUO surgery. Expression of renal fibrosis markers, including fibronectin, Col I, and α-SMA, was increased in UUO mice compared to in WT mice, with *Hif2a* deficiency inhibiting fibrosis marker gene expression (Fig. 5c, d). Masson’s trichrome staining further confirmed that *Hif2a* deficiency alleviated renal fibrosis induced by UUO surgery (Fig. 6a, b). Moreover, changes in BUN levels were consistent with the data of western blot and Masson’s trichrome staining (Fig. 6c). These results indicate that conditional knockout of *Hif2a* in interstitial cells ameliorates UUO-induced renal fibrosis and injury.

### SIRT1/HIF-2α axis is involved in fibrosis in cultured NRK-49F cells

**Fig. 7:**
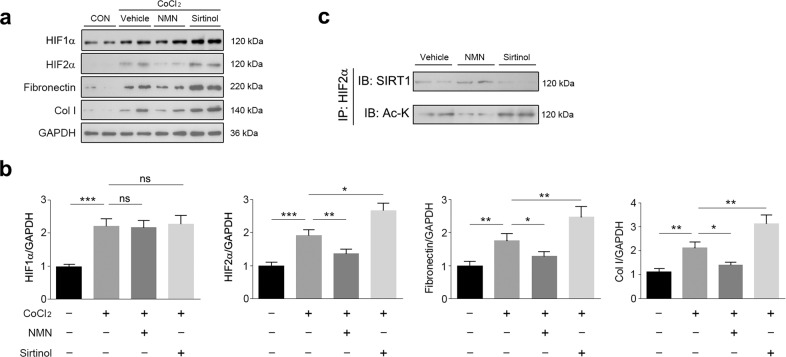
Modulation of SIRT1 activity affects HIF-2α and fibrosis gene expression. **a** NRK-49F cells were treated with CoCl_2_, NMN, Sirtinol as indicated. Cell lysates were prepared for western blot analysis. HIF-1α, HIF-2α, Fibronectin, and Col I were increased by inhibition of SIRT1. GAPDH was used as a loading control. **b** Quantitative analysis of western blot data as shown in **a**. **c** SIRT1 interacts with and deacetylates HIF-2α. NRK-49F cells were treated with NMN, Sirtinol as indicated. Total cell lysates were prepared and subjected to co-immunoprecipitation analysis using an anti-HIF-2α antibody. The immunocomplex was analyzed by western blot. IP: immunoprecipitation. IB: immunoblot. Data are means ± SEM. ns means no significance. *P > 0.05, **P < 0.01, ***P < 0.001.

To further investigate whether SIRT1 regulates kidney fibrosis through HIF-2α, we employed a pharmacological intervention strategy to address this concern. NMN and sirtinol were used, respectively, to activate and inhibit SIRT1 activity. CoCl_2_ was used to induce fibrotic gene expression. By using these chemicals to treat NRK-49F cells, we generated an in vitro fibrosis model in which SIRT1 activity was selectively activated or inhibited (Fig. 7a). The application of CoCl_2_ significantly increased HIF-2α levels, which were further decreased or increased by NMN or sirtinol, respectively (Fig. 7a, b). These changes in HIF-2α level were accompanied by CoCl_2_-induced increases in the occurrence of fibrosis markers, such as fibronectin and Col I. Treatment with NMN attenuated CoCl_2_-induced fibronectin and Col I, while treatment with sirtinol further increased the protein levels of fibronectin and Col I (Fig. 7a, b). As for HIF-1α, it was increased by CoCl_2_; however, it was not further altered by NMN or sirtinol (Fig. 7a, b). The direct interaction of SIRT1 with HIF-2α was bolstered by NMN and weakened by sirtinol (Fig. 7c). Consequently, acetylated HIF-2α levels were decreased by NMN and increased by sirtinol (Fig. 7c). These results suggest that the renoprotective roles of SIRT1 against renal fibrosis are likely due to inhibition of HIF-2α.

## Discussion

SIRT1 is involved in various critical biological processes, including metabolism, oxidative stress, hypoxic stress resistance, DNA repair, and aging. Recently, the antifibrotic function of SIRT1 has been reported in different organs, such as the liver, skin, and breasts^21–23^. In the kidney, SIRT1 is abundantly expressed in interstitial cells of the renal inner medulla, where it plays an important protective role against oxidative stress^6^. In addition, SIRT1 has been shown to alleviate kidney fibrosis^6^. It should be mentioned that these findings were observed in systemic heterozygous SIRT1 knockout mice (*Sirt1*^+/−^)^6^. Renal interstitial cells are the main cells responsible for constructing ECM and thus, play pivotal roles in the development of renal fibrosis. For this reason, in this study, we generated *Sirt1* CKO mice in which *Sirt1* was deleted specifically in renal interstitial cells. Our data showed that *Sirt1* CKO resulted in an increase in the severity of renal damage and fibrosis caused by UUO, which further confirmed the importance of SIRT1 in renal pathology.

In line with our findings, several studies have shown that SIRT1 activation ameliorates kidney damage and fibrosis in various disease models, including ischemia reperfusion-induced acute kidney injury^24^, diabetic nephropathy^25–27^, UUO surgery^6^, and renal aging^28^. Surprisingly, it has also been shown that blockage of SIRT1 by sirtinol attenuates renal fibrosis in obstructive nephropathy^29^. This discrepancy is likely due to the application of sirtinol, which may target other substrates besides SIRT1.

Recently, a growing body of evidence has proposed that the underlying molecular mechanisms for SIRT1 could potentially protect against renal fibrosis. It has been speculated that, in these proposed mechanisms, SIRT1 regulates the expression and/or activity of multiple substrates including TGF-β/Smad3, CTGF, STAT3, HIF-1α, and MMPs^10,30–33^. In our study, we found that SIRT1 deficiency-induced upregulation of HIF-2α expression in the kidneys. In cultured cells, SIRT1 activation resulted in a decrease in HIF-2α expression. These data suggest that SIRT1 is a negative regulator of HIF-2α. Further supporting this notion, an earlier report found an inverse correlation between SIRT1 and HIF-1α in both young and aged kidneys^28^. This report also stated that SIRT1 was shown to inhibit HIF-1α activity and protect the kidneys from damage in aged mice^28^. Consistent with this, another study indicated that deacetylation of HIF-1α by SIRT1 inhibits HIF activity in multiple mouse tissues and cell lines^34^. However, it has also been shown that SIRT1 directly deacetylates HIF-2α, thus stimulating its activity during hypoxia^35^. This discrepancy may be due to different procedures performed on animal subjects, such as UUO surgery versus hypoxia. The differences in conformational structures of HIF-1α and HIF-2α may affect their interactions with SIRT1, which could also explain these contrary observations.

The reverse correlation between SIRT1 and HIF-2α suggests that SIRT1 is a negative regulator of HIF-2α. Moreover, increased HIF-2α levels might be responsible for SIRT1 deficiency-induced severe renal fibrosis. To examine this issue, we bred *Hif2a* CKO mice with *Hif2a* deleted specifically in their renal interstitial cells. Indeed, UUO-induced renal fibrosis was greatly attenuated in *Hif2a* CKO mice, indicating that HIF-2α might promote the development of renal fibrosis and CKD. Renal tissue hypoxia is considered a probable start event and final common pathway in the occurrence and development of various CKD conditions. HIFs are master mediators in the hypoxia response of virtually all cells and tissues^36^. HIF-1 and HIF-2 are major hypoxia-inducible factors across different species, including humans, mice, and rats^36^. HIF-1α and HIF-2α activate transcription of a wide range of target genes with separate but overlapping between the two factors^37^. There are controversial findings of HIFs in the pathophysiology of CKD. Previous studies have shown that the accumulation and stimulation of HIF have renoprotective effects in various kidney disease models^38,39^. Some other pieces of evidence indicate that improper and extended activation of HIFs induces the development of renal fibrosis and CKD^40–43^. Therefore, the precise roles that HIFs play in CKD, which may be determined by different HIF expression profiles, are still under debate.

In summary, our study demonstrates that ablation of *Sirt1* in renal interstitial cells aggravates UUO-induced kidney fibrosis and damage and that the renoprotective role of SIRT1 may be due, at least in part, to its ability to enhance HIF-2α expression and downstream signal transduction. This study reveals the protective role of the SIRT1/HIF-2α axis in kidney fibrosis and CKD, which may provide a new target for the treatment of renal fibrosis and CKD.

## Materials and methods

### Generation of renal interstitial cell conditional knockout (CKO) mice

*Sirt1*^floxed/+^, *Hif2a*^floxed/floxed^, and Tenascin-C Cre (TNCCreER) mice were kindly provided by Prof. Chuanming Hao. Mice were housed in a room with constant temperature (20–23 °C) and humidity (50–60%) inside the animal facility of Nantong University with a 12-h light/dark cycle and allowed free access to standard rodent chow and water. *Sirt1* conditional knockout (CKO) mice were bred by crossing *Sirt1*^floxed/+^ mice (*Sirt1*^floxed/+^ mice in the C57BL/6J background) with TNCCreER mice (TNCCreER mice in B6 background). Cre recombinase was induced in 5-week-old mice by i.p. injection of tamoxifen (20 mg/kg; Sigma Aldrich) for 5 consecutive days. All animal study protocols were approved by the Institutional Animal Care and Use Committee of Nantong University. The procedures for breeding *Hif2a* CKO mice were similar to those used to breed for *Sirt1* CKO mice.

### UUO surgery

8-week-old male mice were anesthetized with 5% chloral hydrate at a dose of 10 mg/kg body weight. Their left ureters were exposed via a lateral incision and ligated with two separate silk ties, level with the lower pole of the kidney. Mice were euthanized 10 days after the surgeries. The obstructed kidneys were collected and assessed for gene expression and fibrosis.

The sample size was six for each group and no animals were excluded. No randomization was used. The researchers were not blinded to the experiments. The animal protocols were approved by the Animal Care and Use Committee of Nantong University and the Jiangsu Province Animal Care Ethics Committee.

### Cell culture

NRK-49F cells were cultured in high glucose in Dulbecco’s modified Eagle’s medium (Gibco) medium containing 10% fetal bovine serum (Gibco), penicillin (100 IU/ml), and streptomycin (100 μg/ml). Cells were maintained at 5% CO_2_ and 37 °C. To induce hypoxia and fibrosis, cells were incubated with 100 μM CoCl_2_ for 24 h. All cell lines were authenticated by morphologic observation under microscopy and tested by short tandem repeat profiling. PCR was used to exclude mycoplasma contamination.

### Co-immunoprecipitation

NRK-49F cells were treated with nicotinamide mononucleotide (NMN) or sirtinol as indicated in the figure legend for 24 h. Cells were then harvested and subjected to co-immunoprecipitation analysis with a commercial kit (Pierce) according to the manufacturer’s instructions, using an antibody against HIF-2α. The resulting immune complexes were analyzed by western blotting.

### Western blotting

The procedures for western blot analysis were described elsewhere^44^. Kidney tissues and cells were subjected to western blot analysis using standard procedure. After blocking nonspecific binding with 5% nonfat milk in phosphate-buffered saline solution (PBST) for 1 h at room temperature, cell membranes were incubated with primary antibody overnight at 4 °C. Then, they were immunoblotted with antibodies against SIRT1 (Abcam; ab18239), fibronectin (Sigma; CP70), collagen I (Novus; NB600-408), α-SMA (Abcam; ab7817), HIF-1α (Cell Signaling Technology; #36169), HIF-2α (Novus; NB100-122), Ac-K (Cell Signaling Technology; #9441), and GAPDH (Abcam; ab8245), as well as the appropriate secondary antibodies. Band intensity was captured using a chemiluminescence reagent (WBKLS0100, Millipore) with a GE ImageQuant LAS 4000. Image J software was used to analyze the densitometry of the bands.

### RNA extraction and qRT-PCR

The normal procedures were described previously^45^. Total RNA was extracted from renal tissues using TRIzol reagent (Invitrogen). Reverse transcription was performed using a first-strand cDNA reverse transcription kit (Takara, RR047A). Real-time PCR was performed using an SYBR Premix Ex Taq II (Tli RNase H Plus) kit according to the manufacturer’s instructions (Takara, RR820). The primers used were as follows: mouse *Sirt1* forward 5’-GCA ACA GCA TCT TGC CTG AT-3’ and reverse 5’-GTG CTA CTG GTC TCA CTT-3’, *Hif2a* forward 5’-GTG ACC CAA GAC GGT GAC AT-3’ and reverse 5’-TCC CAA AAC CAG AGC CGT TT-3’; *Fn1* forward 5’-ATG TGG ACC CCT CCT GAT AGT-3’ and reverse 5’-GCC CAG TGA TTT CAG CAA AGG-3’; *Col1a1* forward 5’-GAG AGG TGA ACA AGG TCC CG-3’ and reverse 5’-AAA CCT CTC TCG CCT CTT GC-3’; *Acta2* forward 5’-GGA GAA AAT GAC CCA GAT T-3’ and reverse 5’-GAG TCC AGC ACA ATA CCA G-3’; *Tgfb1* forward 5’-TGG CGA GCC TTA GTT TGG A-3’ and reverse 5’-TCG ACA TGG AGC TGG TGA AA-3’; 18S forward 5’-CGC CGC TAG AGG TGA AAT TCT-3’ and reverse 5’-CAT TCT TGG CAA ATG CTT TCG-3’.The expression for the mRNA of interest was calculated with a comparative method (2^−ΔΔCT^) normalized to 18S.

### Histology and morphometry

Kidneys were removed and immediately fixed with 4% paraformaldehyde for 2 h at room temperature. The 2–3 μm sections were cut from paraffin-embedded kidney tissues. Sections were subject to Masson-trichrome staining, Sirius red staining, or immunofluorescence analysis for histology analysis.

### BUN assays

Serum blood urea nitrogen (BUN) levels were measured using the UREA KIT (liquid; UV-GLDH method; Shanghai Kehua Bioengineering Co., Ltd.) according to the manufacturer’s instructions. Briefly, 20 μl serum samples were mixed with the Enzyme Solution and incubated at 37 °C for 10 min. Absorbance at 640 nm was recorded with a microplate reader (BioTek, USA). BUN levels were calculated by using the equation as following: BUN level (mM) = (A_sample_ – A_blank_)/(A_standard_ – A_blank_) × standard concentration (mM).

### Statistical analysis

Results were analyzed using a paired Student’s *t* test for comparisons between 2 groups or one-way analysis of variance for comparisons between more than 2 groups. *P* < 0.05 was considered statistically significant. Data are expressed as means ± SEM.
